# Early inner plexiform layer thinning and retinal nerve fiber layer thickening in excitotoxic retinal injury using deep learning-assisted optical coherence tomography

**DOI:** 10.1186/s40478-024-01732-z

**Published:** 2024-02-01

**Authors:** Da Ma, Wenyu Deng, Zain Khera, Thajunnisa A. Sajitha, Xinlei Wang, Gadi Wollstein, Joel S. Schuman, Sieun Lee, Haolun Shi, Myeong Jin Ju, Joanne Matsubara, Mirza Faisal Beg, Marinko Sarunic, Rebecca M. Sappington, Kevin C. Chan

**Affiliations:** 1 Wake Forest University School of Medicine, 1 Medical Center Blvd, Winston-Salem, NC 27157 USA; 2 Wake Forest University Health Sciences, Winston-Salem, NC USA; 3 Translational Eye and Vision Research Center, Wake Forest University School of Medicine, Winston-Salem, NC USA; 4 School of Engineering Science, Simon Fraser University, Burnaby, BC Canada; 5 Department of Ophthalmology, NYU Grossman School of Medicine, NYU Langone Health, New York University, New York, NY USA; 6 Department of Ophthalmology, SUNY Downstate Medical Center, Brooklyn, NY USA; 7 Center for Neural Science, College of Arts and Science, New York University, New York, NY USA; 8 Department of Biomedical Engineering, Tandon School of Engineering, New York University, Brooklyn, NY USA; 9 Wills Eye Hospital, Philadelphia, PA USA; 10 Department of Biomedical Engineering, Drexel University, Philadelphia, PA USA; 11 Neuroscience Institute, NYU Grossman School of Medicine, NYU Langone Health, New York University, New York, NY USA; 12 Department of Ophthalmology and Visual Sciences, The University of British Columbia, Vancouver, BC Canada; 13 Mental Health and Clinical Neurosciences, School of Medicine, University of Nottingham, Nottingham, UK; 14 Department of Statistics and Actuarial Science, Simon Fraser University, Burnaby, BC Canada; 15 Institute of Ophthalmology, University College London, London, UK; 16 Department of Medical Physics and Biomedical Engineering, University College London, London, UK; 17 Department of Radiology, NYU Grossman School of Medicine, NYU Langone Health, New York University, New York, NY USA

**Keywords:** Deep learning, Excitotoxicity, *N*-methyl-d-aspartate, Optical coherence tomography, Retinal thickness, Transfer learning

## Abstract

Excitotoxicity from the impairment of glutamate uptake constitutes an important mechanism in neurodegenerative diseases such as Alzheimer’s, multiple sclerosis, and Parkinson's disease. Within the eye, excitotoxicity is thought to play a critical role in retinal ganglion cell death in glaucoma, diabetic retinopathy, retinal ischemia, and optic nerve injury, yet how excitotoxic injury impacts different retinal layers is not well understood. Here, we investigated the longitudinal effects of N-methyl-D-aspartate (NMDA)-induced excitotoxic retinal injury in a rat model using deep learning-assisted retinal layer thickness estimation. Before and after unilateral intravitreal NMDA injection in nine adult Long Evans rats, spectral-domain optical coherence tomography (OCT) was used to acquire volumetric retinal images in both eyes over 4 weeks. Ten retinal layers were automatically segmented from the OCT data using our deep learning-based algorithm. Retinal degeneration was evaluated using layer-specific retinal thickness changes at each time point (before, and at 3, 7, and 28 days after NMDA injection). Within the inner retina, our OCT results showed that retinal thinning occurred first in the inner plexiform layer at 3 days after NMDA injection, followed by the inner nuclear layer at 7 days post-injury. In contrast, the retinal nerve fiber layer exhibited an initial thickening 3 days after NMDA injection, followed by normalization and thinning up to 4 weeks post-injury. Our results demonstrated the pathological cascades of NMDA-induced neurotoxicity across different layers of the retina. The early inner plexiform layer thinning suggests early dendritic shrinkage, whereas the initial retinal nerve fiber layer thickening before subsequent normalization and thinning indicates early inflammation before axonal loss and cell death. These findings implicate the inner plexiform layer as an early imaging biomarker of excitotoxic retinal degeneration, whereas caution is warranted when interpreting the ganglion cell complex combining retinal nerve fiber layer, ganglion cell layer, and inner plexiform layer thicknesses in conventional OCT measures. Deep learning-assisted retinal layer segmentation and longitudinal OCT monitoring can help evaluate the different phases of retinal layer damage upon excitotoxicity.

## Introduction

The neurotransmitter glutamate serves as a major excitatory neurotransmitter for sensory transmission in the retina. Among the glutamate receptors, the N-methyl-D-aspartate (NMDA) receptor is the primary receptor involved in calcium influx into the neurons upon binding [1, 2]. In the setting of increased extracellular glutamate concentrations in neurodegenerative diseases such as Alzheimer’s, multiple sclerosis, and Parkinson’s disease, the excessive activation of NMDA receptors leads to neuronal cell death via cytochrome c, nitric oxide, p38 mitogen-activated protein kinase, and other pathways [3, 4]. Similarly, excitotoxicity is an important mechanism identified in ocular disease processes including glaucoma, diabetic retinopathy, retinal ischemia, and optic nerve injury [3]. Previous ex vivo and clinical studies have shown that overstimulation of the NMDA receptors leads to excitotoxic retinal injury that impairs retinal morphology and visual function [5–7]. In the inner retina, earlier studies showed the susceptibility of the retinal ganglion cells (RGCs) to NMDA-induced excitotoxicity [1, 3, 4]. However, its vulnerability is highly selective and dependent on distinctive RGC cell types [4, 8]. The RGC degeneration can be differentiated at the subcellular level among dendrites, somas, axons, and synapses [4]. The glutamate receptors are also preferentially concentrated in the inner and outer plexiform layers, which hints at the early involvement of retinal injury beyond the ganglion cell layer (GCL). Recent cross-sectional studies have revealed neurotoxicity-induced alternations in the retinal nerve fiber layer (RNFL), inner plexiform layer (IPL), and inner nuclear layer (INL) in both glaucoma patients [9–11] and glaucoma animal models [12]. However, little is known about their longitudinal pathological profiles across multiple retinal layers after neurotoxicity influx. Since damage to the central nervous system including the retina remains irreversible, understanding the neuropathological cascades after excitotoxic retinal injury is important for improving disease monitoring and unveiling potential targets for earlier and more precise intervention to slow down or halt disease progression. In this study, we aim to address this knowledge gap by investigating the longitudinal effects of excitotoxic retinal injury on degenerative events in different retinal layers using an in vivo experimental rat model. We hypothesize that NMDA-induced excitotoxicity leads to distinct and dynamic patterns of neurodegeneration across retinal layers.

Changes in retinal layer thickness have been used as a surrogate biomarker to reflect excitotoxic retinal injury in various histological animal studies ex vivo. However, histology suffers from tissue shrinkage during destructive processing and is prone to variability when using different samples to evaluate pathological changes across time. Optical coherence tomography (OCT) offers non-invasive, high-resolution 3D volumetric representations of the anatomical structure in the neurosensory retina. This allows tracking of the retinal thicknesses in various clinical ocular conditions in vivo, including glaucoma and diabetic retinopathy [13–15]. OCT has also been used for in vivo evaluation of total retinal thickness changes upon excitotoxic retinal injury in preclinical animal models [16]. The thickness of the ganglion cell complex combining the RNFL, GCL, and IPL has been proposed as a potential OCT imaging biomarker of glaucomatous degeneration in the context of disease progression [14, 17–19]. However, how these individual layers change over the course of the disease has not been well characterized, partly because of the difficulties in separating between these layers reliably, in particular the GCL and IPL. Accurate retinal sublayer thickness estimation requires unbiased whole-volume segmentation of multiple retinal layers. Conventional parameterized automatic retinal layer segmentation approaches such as GraphCut-based methods [20, 21] require extensive parameter tuning, suffer from long and memory-intensive processing time, and are sensitive to noise and local intensity shift. In contrast, recent advancements in deep learning-based automatic segmentation enable more efficient, accurate, and robust segmentation [22–26]. To this end, we have developed a deep learning-based retinal layer segmentation framework that has undergone extensive validation on human retinal OCT data across multiple devices and clinical conditions [27–29]. On the other hand, there are limited preclinical applications of deep learning-based retinal layer segmentation frameworks available for non-human studies, mainly due to the heterogeneity in retinal morphology across different species and strains, variability across OCT imaging systems, limited samples for training from existing individual studies, and the labor-intensive processes to generate high-quality ground truth for training deep learning models [30]. Here, we propose to refine our deep learning-assisted retinal layer segmentation framework for clinical studies and implement the integration of weakly supervised transfer learning [27] based domain adaptation [29] and pseudo-labeling [31] approaches into our analysis pipeline to facilitate preclinical, longitudinal multi-layer retinal thickness estimation in our experimental rat model for this project.

## Materials and methods

### Experimental animal modeling and data acquisition

#### Animal preparation for excitotoxic retinal injury

A total of 9 adult Long Evans rats were included in this study. Excitotoxic retinal injury was induced through a single intravitreal injection of NMDA solution into the right eye. The contralateral left eye did not receive any injection and served as an internal control. For intravitreal injection, the animals were anesthetized with an intraperitoneal injection of ketamine and xylazine cocktail at the dose of 80 mg/kg and 8 mg/kg respectively. The right eye was prepared aseptically using 5% povidone iodine ophthalmic solution and desensitization with 0.5% topical proparacaine hydrochloride ophthalmic solution. Excitotoxic retinal injury was induced through a single 2 µL intravitreal injection of 150 nmol NMDA dissolved in 0.9% saline solution using a 5 µL Hamilton syringe and a 31-gauge RN needle. One drop of 0.3% ciprofloxacin ophthalmic antibiotic solution was applied after the procedure.

#### Retinal imaging through OCT

The spectral-domain OCT (Bioptigen, Inc., Research Triangle Park, NC, USA) was used to image the 1.6 × 1.6 × 1.64 mm^3^ volume along the B-scan (width = 400 pixel), C-scan (depth = 400 pixel), and A-scan (height = 1024 pixel) directions centered on the optic nerve head for both eyes before (day 0) and at 3, 7, and 28 days after unilateral intravitreal NMDA injection. Prior to OCT imaging, the rats were anesthetized with an intraperitoneal injection of ketamine and xylazine. Tropicamide ophthalmic solution (1%) was used to dilate the pupil. Eye drops containing sodium carboxymethylcellulose were applied to neutralize the corneal curvature and keep the cornea hydrated during retinal imaging [32].

### OCT image processing and analysis pipeline

**Fig. 1:**
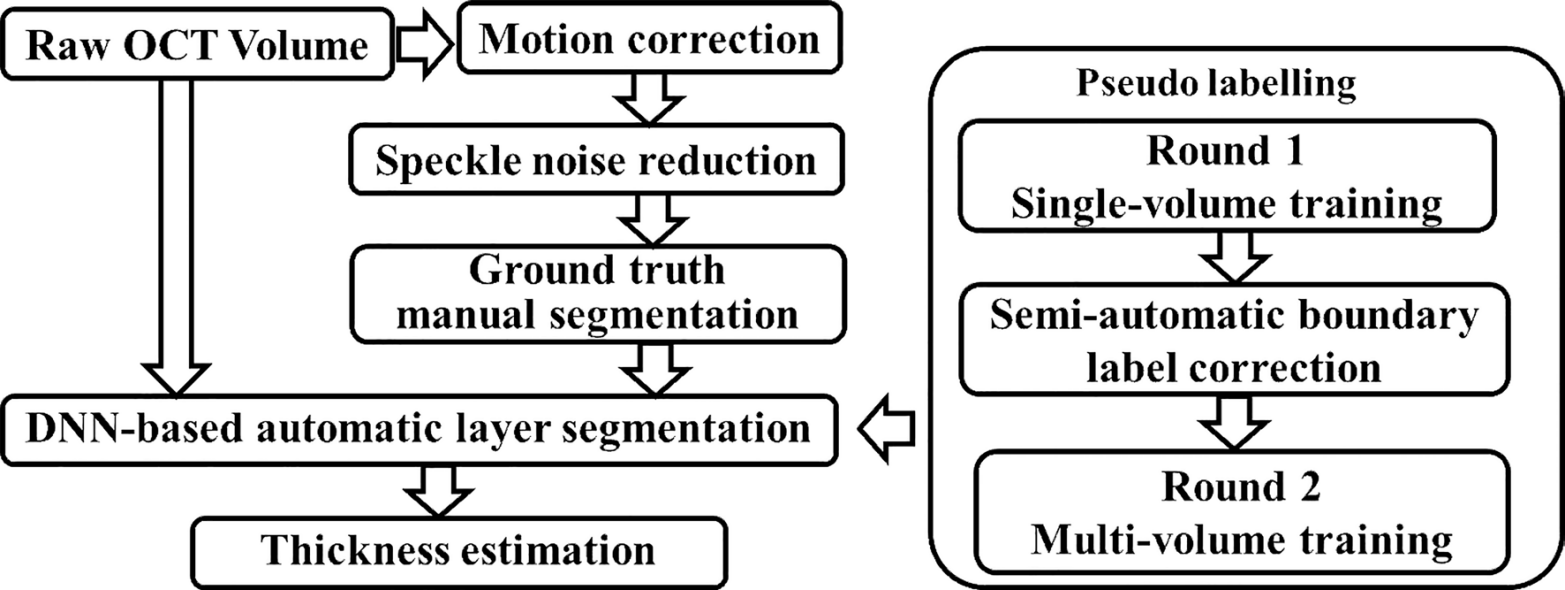
Schematic diagram of the end-to-end processing pipeline for the OCT-based automatic layer segmentation and layer-specific thickness estimation framework

This section describes the end-to-end OCT image processing and analysis pipeline that we developed for measuring the longitudinal retinal layer changes in preclinical rodent experiments. Figure 1 shows the schematic diagram of our deep learning-assisted retinal layer segmentation and thickness estimation framework. Specific procedures are described as follows:

#### Preprocessing

##### Axial motion correction

**Fig. 2:**
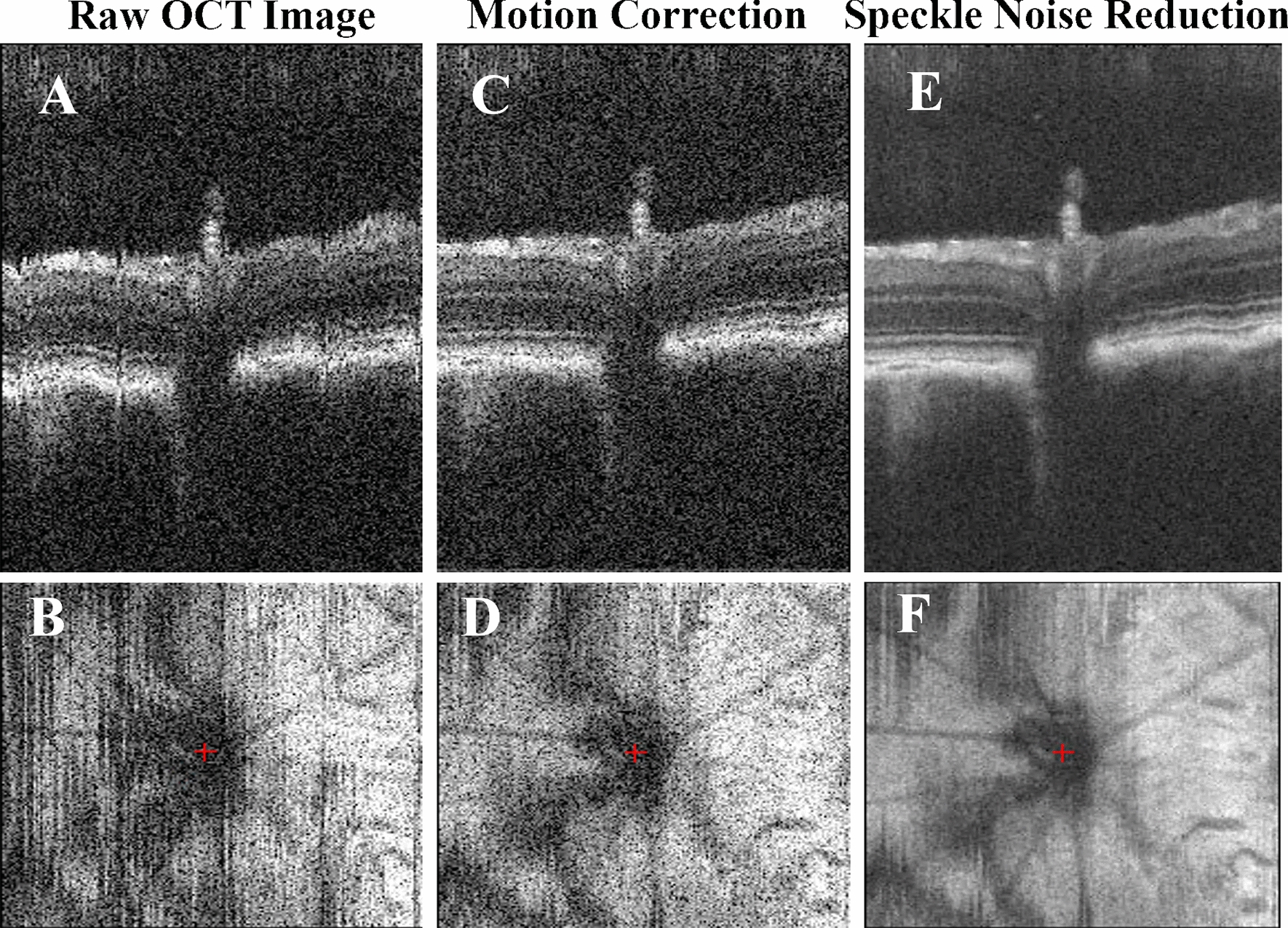
Representative stepwise results of the preprocessing pipeline in OCT. (Top) C-scan view, or slow-scan view across all B-scans in the volume; (Bottom) *En-face* view. **A, B** Raw retinal OCT volume; **C, D** motion-corrected volume along the axial direction; **E, F** speckle noise-reduced volume after the bounded-variation smoothing step

The axial motion during image acquisition was corrected by registering each pair of adjacent B-scans and minimizing the cross-correlation (Fig. 2C, D). A polynomial curve fitting was included in the equation to preserve both the natural curvature of the retina and the scanning angle during acquisition [13]. The motion-corrected OCT images were fed into the next step for 3D noise reduction for visualization and manual labeling.

##### Speckle noise reduction

The speckle noise of the OCT signals was reduced using the edge-preserving denoise algorithm via 3D bounded-variation smoothing [13] to provide better visualizations of the raw OCT images with higher signal-to-noise ratio (SNR) and contrast-to-noise ratio (CNR) (Fig. 2E, F). Bounded-variation smoothing [33, 34] can assist manual raters in identifying retinal boundaries accurately and generating layer segmentation as ground truth labels. The original raw OCT data and the manually delineated and curated retinal layer segmentation labels were used to train the deep learning-based auto-segmentation algorithm without speckle denoising.

#### Deep learning-assisted retinal layer segmentation

To investigate how the injured retinal layers progressed with NMDA-induced excitotoxicity, we used supervised deep learning-assisted retinal layer segmentation on the retinal OCT data. The retinal layers were first manually segmented on each 2D B-scan of two independent OCT volumes acquired from healthy rat eyes in order to serve as the initial training data for automatic segmentation. A total of ten retinal layers were manually segmented, from the combined inner limiting membrane and retinal nerve fiber layer (ILM-RNFL), to the GCL, IPL, INL, outer plexiform layer (OPL), outer nuclear layer (ONL), external limiting membrane (ELM), photoreceptor layer (PRL), retinal pigment epithelium (RPE), and Bruch’s membrane (BM). Automatic retinal layer segmentation was achieved using the LF-UNet [27, 28], which is an anatomical-aware cascaded deep learning-based retinal OCT segmentation framework that has been validated on human retinal OCT data. In order to improve the efficiency and generalizability of the LF-UNet segmentation framework when training with a small, labeled dataset, two techniques were applied: (1) composited transfer learning [27] based domain adaptation [35], and (2) pseudo-labeling [31].

Firstly, optimized model parameter initialization and fast convergence were achieved through transfer learning and domain adaptation. Among the two OCT volumes with ground truth manual segmentation, B-scans from one OCT volume were used as training data, and B-scans from the other volume were regarded as the validation set. Transfer learning was used to initialize the parameter of all the feature extraction layers in the neural network of the LF-UNet deep learning model, significantly reducing the need for training data. These model parameters were pre-trained using a segment task on the human retinal OCT data [27]. The last segmentation layer is a fully connected pixel-wise classification layer, and the model parameters in the convolutional layers before the last segmentation layer were preserved and frozen. The model parameters in the last segmentation layer were then updated during the initial rounds of training epochs to transfer the parameters to the retinal layers of the rat OCT data. After initial convergence, the model parameters in the remaining convolutional layer were then unfrozen and updated to adapt to the image domain of our rat OCT data. Data augmentation was implemented to increase the model’s capability to capture a larger range of anatomical variations. This included random translations of pixels between the range of 0–20 pixels along both axial and lateral directions, random rotations of angle between 0 and 30 degrees, random crop and zoom for up to 25%, and random flip with 50% possibility along the lateral direction (i.e. with vertical flip only to reflect the nature of retinal anatomy).

**Fig. 3:**
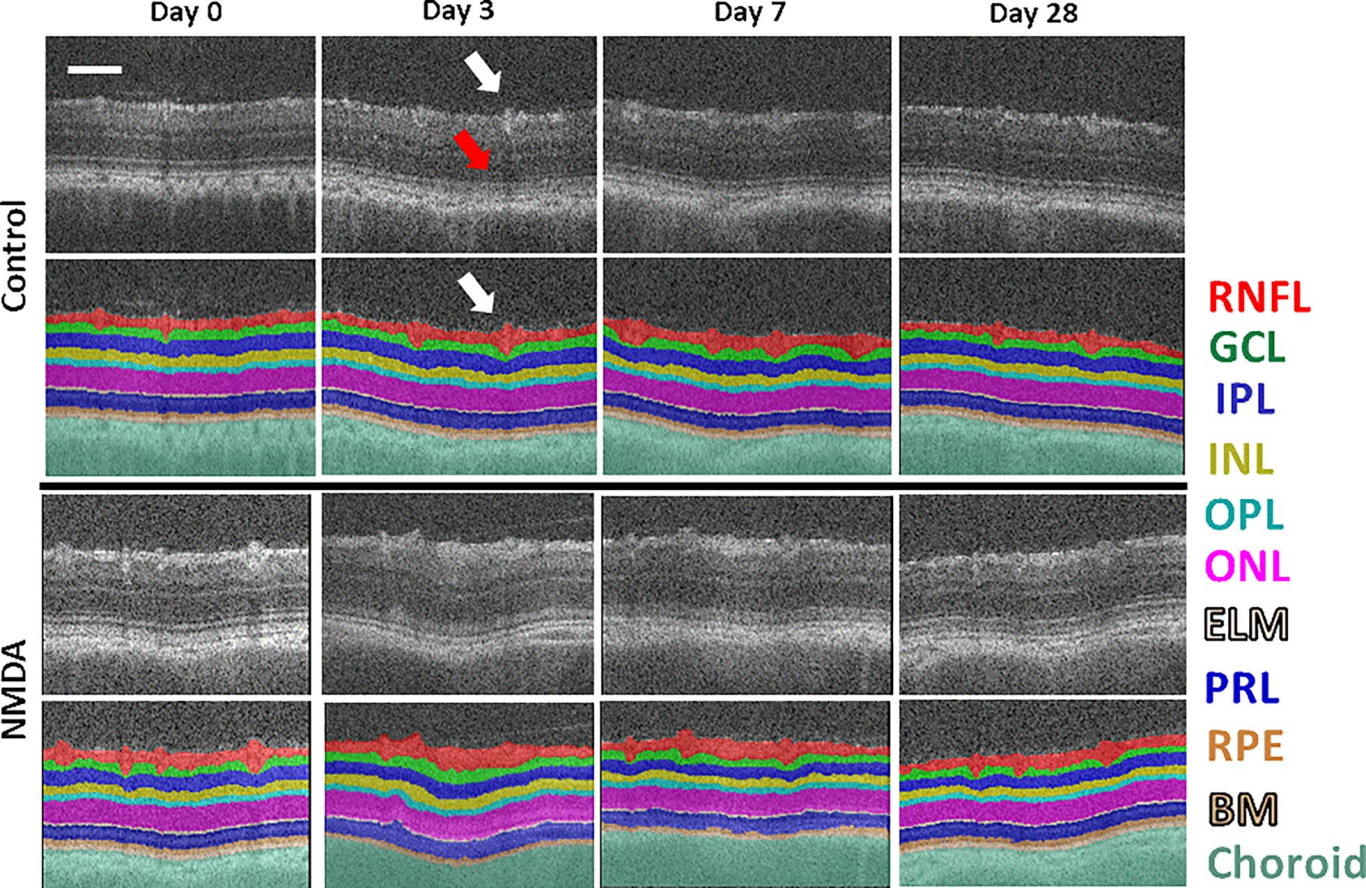
Representative images of automatic retinal layer segmentation from the control eye that did not receive any injection (top two rows) and the experimental eye that received intravitreal NMDA injection (bottom two rows). For each retinal OCT scan, the following ten layers were segmented and measured: combined inner limiting membrane and retinal nerve fiber layer (ILM-RNFL), ganglion cell layer (GCL), inner plexiform layer (IPL), inner nuclear layer (INL), outer plexiform layer (OPL), outer nuclear layer (ONL), external limiting membrane (ELM), photoreceptor layer (PRL), retinal pigment epithelium (RPE), and Bruch’s membrane (BM). The choroid is also labeled underneath BM. Scale bar = 100 μm. The auto-segmentation model was able to follow the blood vessel boundaries in the RNFL layer (white arrows) while avoiding the projection artifacts in the outer retinal layers (red arrow)

A pseudo-labeling approach was then applied to further improve the segmentation accuracy and generalizability of the model with additional unlabeled OCT data. Initially, a total of five out of nine OCT volumes that were acquired at baseline (i.e. day 0 before intravitreal injection) were automatically segmented using the fine-tuned LF-UNet segmentation model. The automatic segmentation results were then combined with the two manually segmented OCT volumes as the intermediate training data containing seven volumes with pseudo-ground truth labels. These intermediate training data then underwent a second round of training. The final trained model was applied to all the axial motion-corrected longitudinal OCT volumes (Fig. 3).

#### Retinal thickness measurements

The layer-specific thickness map was calculated for each retinal scan on the axial motion-corrected OCT volumes without speckle denoising. Firstly, the resultant retinal layer segmentation in each OCT volume was converted from the pixel-wise dense label representation to the surface maps of retinal boundaries. The surface level at each retinal boundary was represented as a 2D point cloud. The thickness of each retinal layer was then calculated at each voxel on the surface map by finding the nearest point between adjacent boundaries using the k-nearest-neighbor search algorithm (k = 1), following the shortest-distance algorithm introduced in the FreeSurfer package [36] similar to the cortical thickness calculation in anatomical brain magnetic resonance imaging. The OCT volumetric data is highly anisotropic, with the lateral resolution (4 µm voxel dimension) lower than the axial resolution (1.6 µm voxel dimension). To ensure accurate thickness estimation, the surface boundary layer distances were first converted to an isotropic sampling space (1.6 µm isotropic) to effectively upsample the lateral resolution. The resultant point-wise retinal surface thickness maps were then converted to their physical dimensions by multiplying the upsampled pixel dimension.

**Fig. 4:**
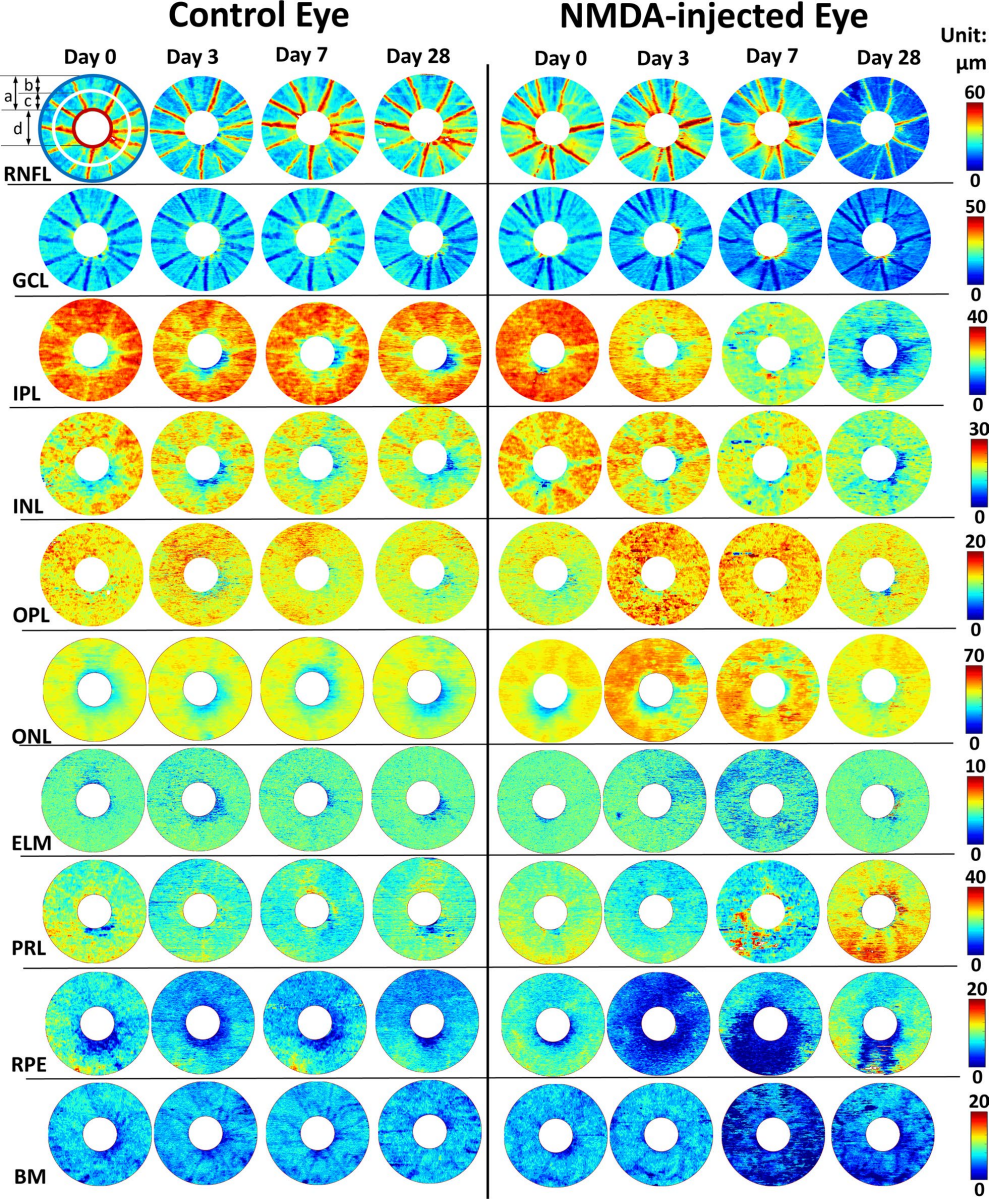
Retinal layer thickness maps in both the NMDA-injected eye (right panel) and the contralateral control eye (left panel) of a representative rat. The images showed gradual thickness changes along 4 time points: Day 0 prior to the unilateral intravitreal NMDA injection, and Day 3, Day 7, and Day 28 after NMDA-induced excitotoxic retinal injury. For each retinal scan, the thickness maps of the following ten retinal layers were measured: ILM-RNFL, GCL, IPL, INL, OPL, ONL, ELM, PRL, RPE, and BM. The region around the optic nerve head was excluded from the downstream analysis. Top left sample illustrated the definition of the full field-of-view (**a**, red-to-blue), peripheral retina (**b**, white-to-blue), and central retina (**c**, red-to-white) for calculating the mean retinal thickness, as well as the optic nerve head mask (d. white central circle)

To further assess the extents of excitotoxic damage in different areas of the retina, we also calculated the mean thickness measurements in both the central and peripheral retinas. The mean retinal thicknesses in the full field-of-view (FOV), and in the central and peripheral retinas were calculated using circular regions of interest (ROI) centered at the optic nerve head as illustrated in the top-left sample image in Fig. 4. The optic nerve head region was excluded from the thickness analysis using a wide margin mask. The mean thicknesses of the total retina, the inner retinal layers (i.e., combination of ILM-RNFL, GCL, IPL, INL, OPL, and ONL), and the outer retinal layers (i.e., combination of ELM, PRL, RPE, and BM) as well as their individual layers were reported.

#### Statistical analysis

A pairwise t-test was conducted to compare the thickness measurements between the NMDA-injected eye and the contralateral uninjured eye at each time point. Multiple comparisons were controlled with false discovery rate = 0.1. Results were considered statistically significant when corrected *p* < 0.05.

## Results

### OCT image preprocessing and retinal layer segmentation

Accurate segmentation of retinal layers in OCT is challenging due to the presence of speckle noise and low contrast in some neuronal layers. To tackle these challenges, this study developed an end-to-end OCT image processing and analysis pipeline for measuring the longitudinal retinal layer changes in preclinical rodent experiments, by extending our previous research on a clinically validated retinal layer segmentation pipeline [13, 27, 28] that was based on the deep convolutional neural network [28] and clinical retinal OCT data [27]. Figure 2 shows the sample raw OCT B-scan images and the results of each preprocessing step. After motion correction, the reduced axial motion artifact led to a smoother representation of the retinal layer boundaries (Fig. 2A–D). This process ensured the preservation of the original anatomical shape for an accurate representation of the layer thickness in the final thickness estimation step. The speckle noise reduction through the bounded-variation smoothing allowed more accurate delineation of ground truth layer segmentation for training the deep learning-based auto-segmentation model. Figure 3 shows the representative images of the OCT B-scans and their automatic retinal layer segmentation from the experimental NMDA-injected eye and the contralateral control eye at each experimental time point using the deep learning-assisted LF-UNet pipeline. The auto-segmentation model was able to follow the blood vessel boundaries in the RNFL layer while avoiding the projection artifacts in the outer retinal layers.

### Longitudinal profiles of retinal degeneration based on layer-specific thickness changes

Figure 4 shows the thickness maps of all 10 segmented retinal layers in both the NMDA-injected eye and the contralateral control eye of a representative animal. In the control eye, no apparent longitudinal thickness change was observed across any retinal layers. In contrast, distinct patterns of layer-specific thickness changes could be observed in the experimental NMDA-injured retina. For instance, within the inner retina (i.e., ILM to ONL), IPL, INL, and RNFL thinning could be observed in the NMDA-injected eye relative to the contralateral control eye at 3, 7, and 28 days post-injection, respectively. Within the outer retina (i.e., ELM to BM), thinning of the photoreceptor and RPE layers could be observed at days 3 and 7 after NMDA injection, whereas the BM thickness reduced at day 7 and 28.

**Fig. 5:**
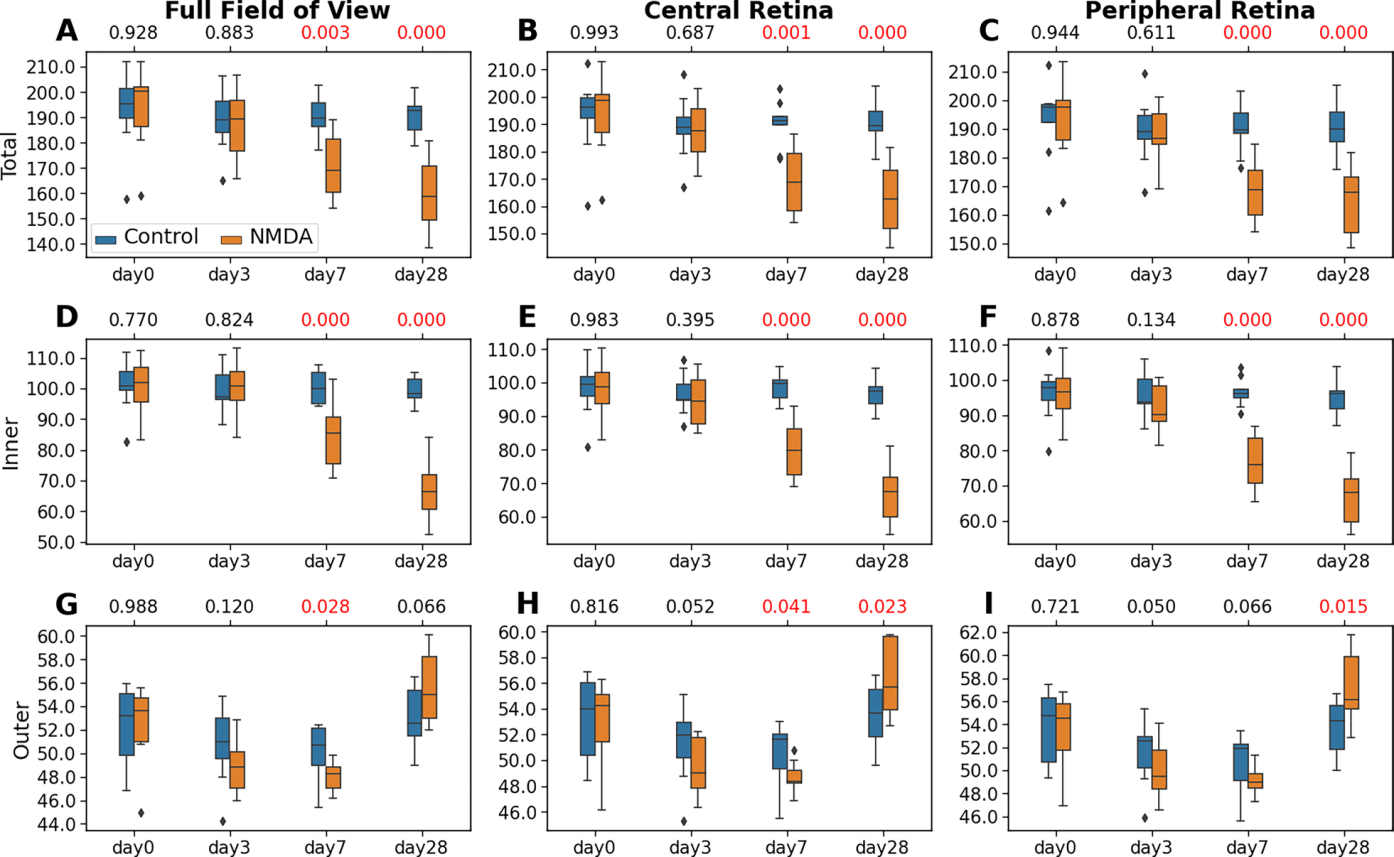
Statistical analyses of the thicknesses of the total retina (**A–C**), inner retina (ILM-ONL) (**D–F**), and outer retina (ELM-BM) (**G–I**), derived from the nine experimental animals. Measurements were made for the full field-of-view (left column), as well as both the central (mid-column) and peripheral retinas (right column). The distributions are represented using box and whisker plots and the outliers are plotted outside of the lines. In descending order, the lines in the plots represent: maximum, third quartile, median, first quartile, and minimum. Retinal thinning was observed in both inner and outer layers of the NMDA-injected eyes, with similar severity between central and peripheral retinas. The longitudinal total retinal thickness reduction in the NMDA-injected eye was mainly contributed to by the inner retina. A pairwise t-test was conducted to compare the thickness difference between the NMDA-injected eye and the contralateral control eye at each time point. Multiple comparisons were controlled with a false discovery rate (FDR) = 0.1. The FDR-corrected p-values were shown at the top of each subplot, with the significant *p* values (< 0.05) shown in red

Figure 5 shows the thicknesses of the total retina (*i.e.*, ILM to BM), inner retina, and outer retina within the full, central, and peripheral regions for all animals. No apparent thickness change was observed in the left uninjured control retina over time. In the NMDA-injected eye, significant total retinal thickness reduction was observed relative to the contralateral eye at day 7 and day 28 in full FOV (Fig. 5A), and both the central (Fig. 5B) and peripheral retinas (Fig. 5C). The main contribution of the reduced total retinal thickness at days 7 and 28 was apparently in the inner retina (Fig. 5D–F), with the full FOV retinal thickness showing an approximately 10.5% and 15.8% decrease, respectively in the NMDA-injected eye relative to the control eye. The outer retina thickness was reduced at day 7 by 3.9%, which then significantly increased at day 28 by 5.5% in the NMDA-injected eye relative to the control eye (Fig. 5G–I).

**Fig. 6:**
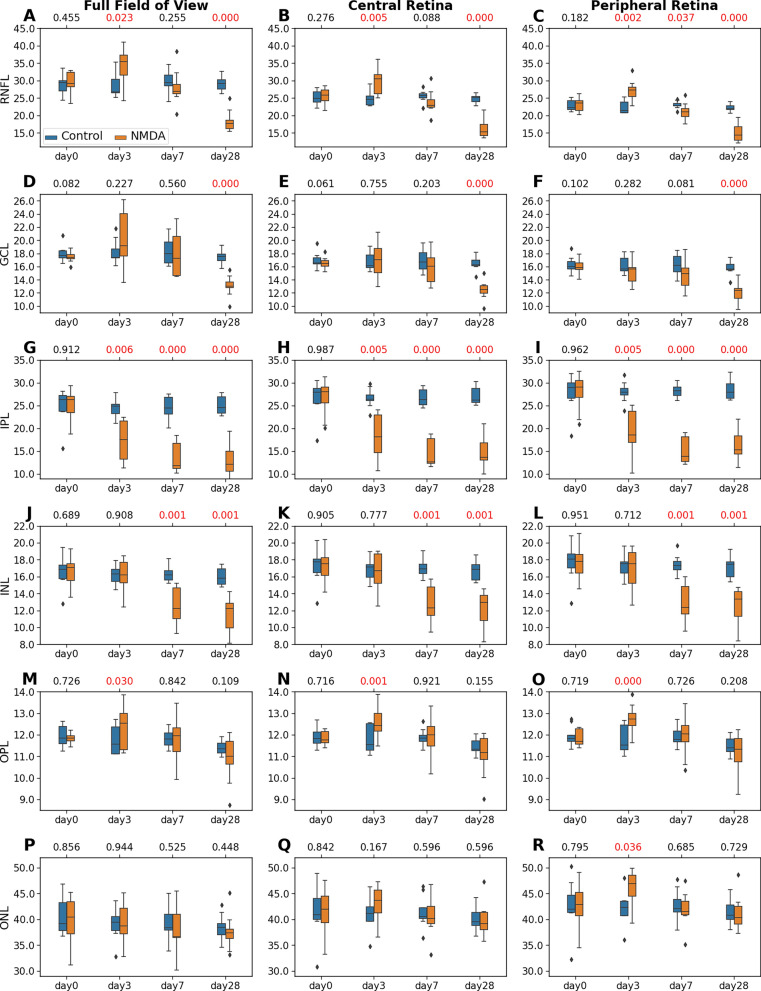
Statistical analyses of the thicknesses of the inner retinal layers including ILM-RNFL (**A–C**), GCL (**D–F**), IPL (**G–I**), INL (**J–L**), OPL (**M–O**), and ONL (**P–R**), derived from the nine experimental animals. The distributions are represented using box and whisker plots and the outliers are plotted outside of the lines. Differential patterns of retinal thickness changes were observed across the inner layers of the NMDA-injected eyes, with similar severity between central and peripheral retinas. A pairwise t-test was conducted to compare the thickness difference between the NMDA-injected eye and the contralateral control eye at each time point. Multiple comparisons were controlled with a false discovery rate (FDR) = 0.1. The FDR-corrected p-values were shown at the top of each subplot, with the significant *p* values (< 0.05) shown in red

**Fig. 7:**
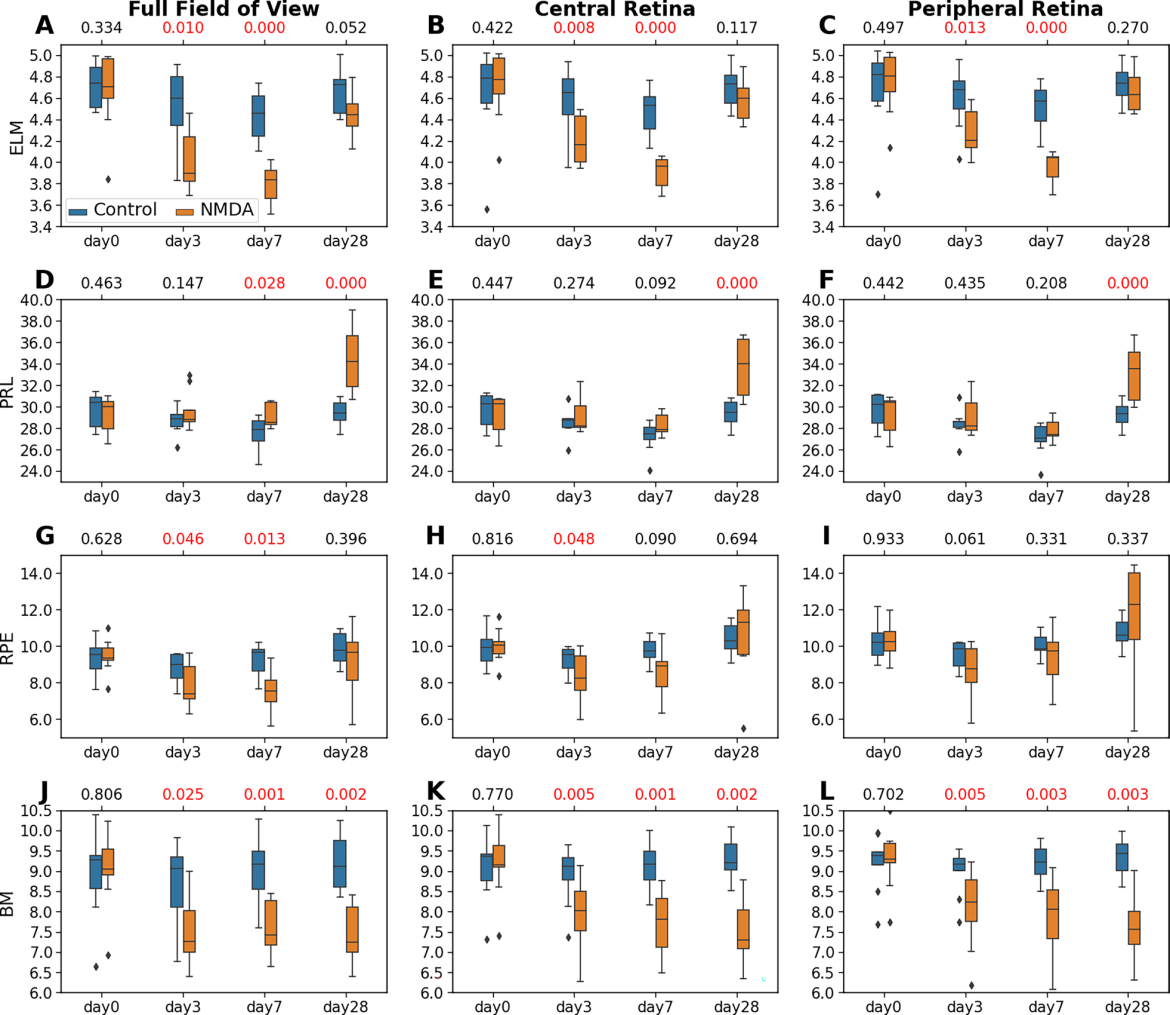
Statistical analyses of the thicknesses of the outer retinal layers including ELM (**A–C**), PRL (**D–F**), RPE (**G–I**), and BM (**J–L**), derived from the nine experimental animals. The distributions are represented using box and whisker plots and the outliers are plotted outside of the lines. Retinal thickness changes were observed in several outer layers of the NMDA-injected eyes, with similar severity between central and peripheral retinas. A pairwise t-test was conducted to compare the thickness difference between the NMDA-injected eye and the contralateral control eye at each time point. Multiple comparisons were controlled with a false discovery rate (FDR) = 0.1. The FDR-corrected p-values were shown at the top of each subplot, with the significant *p* values (< 0.05) shown in red

To further understand the longitudinal effects of NMDA-induced excitotoxicity on the individual retinal layers, we analyzed the thickness of each of the inner retinal layers (*i.e.*, ILM-RNFL, GCL, IPL, INL, OPL, and ONL; Fig. 6) and outer retinal layers (*i.e.*, ELM, PRL, RPE, and BM; Fig. 7), and compared these measures between the NMDA-injected eye and the contralateral control eye. Overall, the full FOV retina (left column), central retina (middle column), and peripheral retina (right column) began with no difference in retinal thickness between the contralateral eyes at baseline (day 0), and then exhibited similar patterns of layer-specific thickness changes in the NMDA-injected eye over time.

Within the inner retina, the ILM-RNFL of the NMDA-injected eye was significantly thicker than that of the contralateral control eye at day 3 (Fig. 6A). The ILM-RNFL thickening was then normalized at day 7 (Fig. 6B) and became significantly thinner than the contralateral eye at day 28 (Fig. 6C). The GCL underwent a similar longitudinal thickness change as in ILM-RNFL, except that the thickness difference was less significant at day 3 (Fig. 6D–F). The IPL (Fig. 6G–I) in the NMDA-injected eye was significantly thinner than the control eye from day 3 to day 28. The INL (Fig. 6J–L) of the NMDA-injected eye was significantly thinner than the control eye at day 7 and day 28. Similar patterns of longitudinal changes were noted in the central and peripheral retinas. No significant between-eye differences were observed in the OPL or ONL thickness except for a slight thickening at day 3 (Fig. 6M–R).

Within the outer retina, the ELM of the NMDA-injected eye became significantly thinner at days 3 and 7, and normalized to the pre-injection thickness at day 28 in both the central and peripheral retinas (Fig. 7A–C). A similar pattern was observed in the RPE layer mainly in the full FOV (Fig. 7G–I). The PRL did not differ between contralateral eyes initially but became thicker in the NMDA-injected eye than the control eye in both the central and peripheral retinas at day 28 (Fig. 7D–F). The BM thickness was significantly reduced starting from day 3 and remained stable until day 28 (Fig. 7J–L).

## Discussion

This study demonstrated the longitudinal effects of excitotoxicity on retinal integrity upon unilateral NMDA injection, using OCT-based deep learning-assisted retinal layer segmentation and thickness monitoring estimation. The retinal thicknesses showed distinct layer-specific temporal patterns, while similar spatial patterns were observed between central and peripheral regions. Within the inner retina, we observed early IPL thinning followed by INL thinning, whereas RNFL initially thickened before normalizing and thinning, implicating the IPL thickness as an early imaging biomarker of excitotoxic retinal degeneration. Within the outer retina, early but slight thinning occurred in ELM, RPE, and BM, followed by normalization of ELM and RPE up to 4 weeks post-NMDA injection, while PRL showed no thinning but delayed thickening at day 28. The distinct temporal patterns of thickness changes across different retinal layers indicated the importance of determining the dynamics of neurodegenerative events for more targeted interventions at different stages of disease progression.

### NMDA-induced retinal excitotoxicity manifested in terms of distinct, layer-specific structural changes during disease progression

Excitotoxicity from glutamate uptake impairment and NMDA overstimulation is believed to play a key role in various retinal pathologies [5–7]. Our study provides a comprehensive framework for quantitative analyses of layer-specific changes in retinal thickness across 28 days of NMDA-induced excitotoxicity in a rat model. Specifically, to investigate the longitudinal changes in retinal layer thickness, we created an end-to-end artificial intelligence (AI)-assisted automatic pipeline to correct retinal axial motions, segment the retinal layers, and calculate the mean retinal layer thicknesses in the central and peripheral retinas. Our results showed that the total retina of the NMDA-injured eye became significantly thinner compared to the contralateral eye from day 7 up to day 28 after intravitreal injection, with a majority of the thickness reduction attributed to the inner retina. Furthermore, individual layer thickness estimation provided a more sensitive and earlier imaging biomarker than the total retinal thickness, inner retinal thickness, and outer retinal thickness, with the first sign of inner retinal degeneration being observed in the IPL at day 3, followed by INL at day 7. This observation appeared consistent with recent histological studies demonstrating early shrinkage of RGC dendrites and presynaptic connections in the IPL before observable RGC and axonal damage in glaucoma and optic nerve injury models [4, 8, 37–41], demonstrating potential clinical relevance of early IPL alteration in glaucoma patients associated with worsening visual function [10, 42]. On the other hand, the ILM-RNFL in the NMDA-injected eye underwent different levels of increases in thickness at day 3, followed by pseudo-normalization at day 7 and significant thinning at day 28 as compared to the contralateral control eye. This temporal pattern of an increase in thickness preceding a decrease in RNFL suggests inflammation before cellular and axonal death. Prior studies have also reported thickening of the inner retina or RNFL due to inflammation [43–45], inflammatory and oxidative stress signaling with NMDA overstimulation [46–48], early inner retina thickening with NMDA overstimulation [49], as well as dendritic shrinkage visible by fluorescence imaging prior to ganglion cell complex thinning in other disease models [50]. Taken together, these findings call for caution in differentiating healthy tissues from pseudo-normalization when interpreting changes in retinal thickness, and the need to take longitudinal measurements when examining OCT scans in retinal diseases.

Glutamate uptake impairment is thought to be a major factor in neurological diseases where excess glutamate in the extracellular compartment leads to excessive activation of NMDA receptors and causes excitotoxic damage to neurons [51]. The NMDA receptor ligand-gated calcium channel contains four subunits (GluN2, GluN3, and two GluN1 subunits) [52]. Different subunits have been shown to play layer-specific roles in the retina. For example, GluN2 has been shown to potentially serve a neuromodulatory role in the IPL, whereas increased expression of GluN2B isoform has been implicated in the degeneration of the RGC layer in glaucoma [53]. RGCs exposed to elevated intraocular pressure increased their susceptibility to glutamate-induced death, and subjecting these cells to both elevated pressure and glutamate led to induction of apoptosis and BAX, suggesting glutamate and increased intraocular pressure together may play a part in the pathogenesis of glaucoma [54–56]. Several clinical studies have suggested RNFL thickness to be useful in the diagnosis and monitoring of glaucoma [57, 58], while others suggested GCL-IPL or IPL thickness alone were more strongly associated with the severity of disease [18, 19]. On the other hand, INL thickness was found to be relatively unaffected in patients with long-standing glaucoma [19]. However, these studies only measured retinal layer thickness at a single time point. It is essential to evaluate the longitudinal effects of excitotoxicity on the retinal cytoarchitecture and functionality in order to unveil and localize the pathological cascades across retinal layers, to guide early disease detection, and to monitor and optimize targeted neuroprotective treatment [59, 60].

Previous studies using ex vivo immunohistochemistry staining of rat retinal samples after NMDA injection have shown retinal layer thinning and apoptotic changes, especially in the GCL, and the severity of neurodegeneration increased with NMDA dosage [61–63]. However, prior histology studies only reported the inner retinal layer degeneration at either day 7 [61, 63] or 14 [62], lacking the ability to evaluate longitudinal effects within the same animals. A recent study introduced in vivo OCT on a chicken model of NMDA-induced retinal injury and reported significant retinal thickness reduction in the IPL, although at a relatively late time point at 14 days after injection [64], and the thickness was derived through manual selection of eight measurement points across the retina. Comparatively, the results of our current study using a rat model showed time-dependent NMDA-induced retinal thickness alterations, especially in the inner retinal layers, across 3 to 28 days after excitotoxic retinal injury. Specifically, our longitudinal thickness analysis revealed significant thinning of the IPL as early as at 3 days post-NMDA injection, with a significant RNFL layer thinning at a later time point (28 days post-NMDA injection) after initial retinal thickening. These observations align temporally with early phases of inflammatory signaling. Furthermore, our study identified NMDA-derived outer retinal layer alterations, indicating a potential neurotoxicity effect towards the outer retina. However, as the outer retinal changes reported are small in the order of 1–2 microns, the axial resolution of the instrument and the accuracy of the algorithm should be taken into account while interpreting the results. Overall, the results of this study provided novel insights about the dynamic and layer-specific patterns of neurotoxicity in the retina. The differential structural changes in retinal thicknesses of the NMDA-injured eyes implicated different pathological processes as well as compensatory mechanisms across retinal layers, which offered an important step to guide further studies to identify the underlying cellular mechanisms at each time point. Last but not least, our findings indicated that OCT with appropriate segmentation protocols could serve as a high-throughput, cost-effective, and non-invasive alternative to complement histological studies. For example, when using histology to assess early mouse retinal changes from 4 h to 7 days after NMDA-induced excitotoxicity, early TUNEL reactivity was found in the INL followed by increased TUNEL reactivity in the GCL and PRL that peaked at 24 h post-NMDA injection [65]. This early neuropathology was accompanied by distinct phases of inflammatory signaling ranging from 24 h to 7 days. The spatiotemporal changes in retinal thickness detected by our AI-assisted OCT imaging generally aligned with these pathological events, but in a non-invasive, in vivo*,* and longitudinal imaging setting within the same cohort of rats. These technological advancements can allow a close monitoring of the disease progression with or without pre- or post-conditioning in order to facilitate testing of causal pathophysiological mechanisms and neurotherapeutic effects with rigor.

### Translational applications of AI-integrated pipeline for automated processing of retinal thickness changes in small animal studies

Recent innovations in AI methods have benefited clinical research substantially. For instance, deep learning-based medical image analysis has been used in ophthalmic big data containing OCT for computer-assisted diagnosis of retinal diseases [22, 66, 67]. In contrast, preclinical animal studies usually involve small sample sizes, hindering the effective adaptation of modern deep learning-based AI applications, which generally require large, labeled samples to train the models accurately. Reverse translation of clinically derived AI methods into preclinical small animal studies may offer a solution to this data availability challenge [30]. A robust automatic retinal layer segmentation pipeline for animal OCT data would significantly improve the processing throughput, measurement repeatability, and analysis accuracy. In this study, we have extended our previously developed and clinically validated deep learning-based automatic retinal layer segmentation framework [27, 28, 68] into adult rat OCT images. It is worth noting that, compared to the human retina, the rat retina lacks a fovea. To this end, the LF-UNet deep learning-based retinal layer segmentation framework that we developed and used in this study was trained on 2D B-scans extracted from the original 3D OCT volume, with a large proportion of 2D B-scans at the non-foveal locations, which ensured that the segmentation model had a good understanding of the general retinal structure across different B-scan locations.

To address the challenges of the lack of standardized retinal layer segmentation labeling, the limited data with ground truth labels of layer segmentation, and the diverse levels of anatomical variations due to experimentally-induced retinal pathology, we have implemented a composition of two techniques, transfer learning [27] based domain adaptation [35] and pseudo-labeling [31], into our existing retinal layer segmentation pipeline, in combination with the data augmentation technique. Firstly, the transfer learning technique used a pretrained model trained from a large number of human retinal data with segmentation labels. Such an approach ensured that the segmentation model parameters were initialized with a good understanding of the general retinal anatomy and OCT image characteristics, significantly reducing the need of training data from rat retinal OCT with ground truth labels in the fine-tuning step. Secondly, during the fine-tuning step, data augmentation was used to introduce random affine transformation to the input data, increasing the range of structural variability even with small training data. The combination of these two steps has shown to allow effective training of AI models in few shots while achieving good performance with few samples [69]. Finally, the pseudo-labeling technique further improved the generalizability of the automatic segmentation model by gradually expanding the training data, so as to propagate and further fine-tune the model parameters with an increased semi-automatic training sample using (pseudo-)ground truth labels from both manual and automatic segmentations. Using this extended framework, we were able to automatically and robustly analyze the neurotoxicity-induced thickness changes across ten retinal layers simultaneously for spatiotemporal assessments.

### Limitations and future directions

The current study focused on the in vivo examination of retinal layer thickness as a surrogate measurement of NMDA-derived excitotoxicity. The primary objective is to leverage a well-established animal model and use AI-based OCT image processing and analysis to facilitate the longitudinal assessment of the neurotoxic effects across layers over 4 weeks. While human and rodent eyes share many similarities, there are also challenges in reverse translation across species such as the size differences, intrinsic structural differences including the lack of fovea in rodents, as well as the differences in OCT devices used for collecting clinical and preclinical OCT data that should be further studied. In the current study, a wide margin mask was used to excuse the optic nerve region from deriving the layer segmentation and estimating the thickness analysis. Given the known anatomical differences of the optic discs between human and rodents, further studies with additional optic nerve labeling would be beneficial to segment the optic nerve head structures and analyze the effects of NMDA-induced excitotoxicity towards the optic disc.

A recent study reported the potential induction of retinal degeneration upon intravitreal normal saline injection in C57BL/6J mice [70]. While our previous study using Sprague–Dawley rats did not show apparent retinal thickness changes upon intravitreal normal saline injection [16], future studies can consider phosphate-buffered saline as the diluent of NMDA instead of normal saline to avoid any potential complications. The translatability and generalizability of the current findings in the NMDA-induced excitotoxic retinal injury model should also be validated in other animal models. When analyzing longitudinal data, different approaches could be used to address the research questions of interest. The current study focuses on assessing the excitotoxic effects of NMDA injection compared to the non-injected contralateral eye at each time point to account for the physiological and age-related changes that may occur in the rat retina. Therefore, we used pairwise group comparisons of the mean retinal layer thicknesses between the injected and control eyes at each time point, which intrinsically accounted for the potential longitudinal retinal layer variations in the contralateral non-injected eye. Future studies may consider repeated measures ANOVAs or other statistical models to examine the overall longitudinal thickness variations for both eyes with larger samples. Further histological studies can also be conducted to confirm the retinal layer boundaries and identify cell-type specific responses underlying the morphological changes detected in the current study. We can also combine non-invasive retinal OCT, brain magnetic resonance imaging, and visual functional assessments to determine the interactions between eye, brain, and behavior in health and disease.

## Conclusion

Using longitudinal OCT monitoring and deep learning-assisted automatic retinal layer segmentation, we demonstrated the pathological cascades of NMDA-induced excitotoxic retinal injury across multiple layers over 4 weeks of experimental period. Thicknesses from individual retinal layers offered more sensitive and specific imaging biomarkers than the combined total retinal thickness, inner retinal thickness, and outer retinal thickness in monitoring retinal injuries, whereas caution is warranted when interpreting the ganglion cell complex combining RNFL, GCL, and IPL thicknesses in conventional OCT measures, as these layers can thicken and thin to different extents and the ganglion cell complex may pseudo-normalize during disease progression. Deep learning-assisted retinal layer segmentation and longitudinal OCT monitoring can help evaluate the different phases of retinal layer changes upon exposure to excitotoxicity***.*** These findings implicate the importance of monitoring the distinct spatiotemporal patterns of neurodegenerative events for guiding more targeted and effective interventions at different stages of disease progression.

## Data Availability

The datasets collected and analysed during the current study are available from the corresponding authors on reasonable request.

## Abbreviations

2D: Two dimensional
3D: Three dimensional
AI: Artificial intelligence
BAX: Bcl-2 Associated X-protein
BM: Bruch’s membrane
CNR: Contrast-to-noise ratio
ELM: External limiting membrane
FDR: False discovery rate
FOV: Field of view
GCL: Ganglion cell layer
ILM: Inner limiting membrane
INL: Inner nuclear layer
IPL: Inner plexiform layer
NMDA: N-methyl-D-aspartate
OCT: Optical coherence tomography
ONL: Outer nuclear layer
OPL: Outer plexiform layer
PRL: Photoreceptor layer
RNFL: Retinal nerve fiber layer
ROI: Region of interest
RGC: Retinal ganglion cell
RPE: Retinal pigment epithelium
SNR: Signal-to-noise ratio
TUNEL: Terminal deoxynucleotidyl transferase dUTP nick end labeling
